# Supplementary material for: Comparing virtual reality, desktop-based 3D, and 2D versions of a category learning experiment
Source: PLoS One. 2022 Oct 6;17(10):e0275119. doi: 10.1371/journal.pone.0275119 (PMC9536585; doi:10.1371/journal.pone.0275119)
Supplement: Attachment — Submitted filename: Response to Reviewers.pdf [file pone.0275119.s001.pdf]

Responses to Reviewers

EVALUATION
It is critically important to show that tasks in a virtual environment function equivalently to tasks in typical desktop 3D and paper-and-pencil 2D tasks. This is because a virtual environment has as much experimental control as the others but is more ecologically relevant, overcoming a common criticism of 3D and 2D tasks. Therefore, the basic premise of the paper is valid, and the results would be of interest to cognitive psychologists and neuroscientists.

However, I have several concerns about the current manuscript, as detailed below. I reserve judgment about the discussion until these are addressed.

1. The introduction should be refocused.
Although the structure of the introduction is reasonable, it obscures the information that is directly relevant to the current project. Currently, the introduction describes the studies that show similar results between virtual and desktop/paper tasks, and then those that show different results. In addition, it describes the benefits of using virtual tasks, including that people enjoy learning tasks virtually, and in some cases, the task is more realistic and therefore more relevant for learning the task. But, the tasks included in the introduction vary widely in terms of embodiment and very few use basic cognitive tasks. I suggest the following changes.

a. The introduction should focus on research that is more similar in terms of basic cognitive processes. Provide more detail about the tasks and the results.

>> We have made extensive revisions to the introduction. We have condensed or cut many of the more peripheral citations, and have made it clearer that there has been no work examining category learning in VR, and thus our work makes a novel contribution in this regard. Despite no directly equivalent papers, we have increased the amount of detail on the most similar work in category learning using common computer presentations.

For the results, evaluate the learning process: initial task learning, learning rate across time, and asymptotic performance.

>> We have included new analyses to assess these variables. They were not included initially because there are few detailed analyses in the existing literature, prohibiting comparison from our data to existing findings.

b. Make a more convincing case for using virtual tasks over traditional tasks; for example, focus on the enjoyment aspect and ecological validity (immersion). These were mentioned but should stand out more. My previous comments highlight the enjoyment argument because that may be the best one to use for a basic cognitive task.

>> In the revised introduction we make clearer that the findings of our study provide evidence about the similarities and differences between VR and traditional computing, from a learning/information access cost perspective. The research is enlightening regardless of the findings: if VR were better, then our study would support its use for learning/optimization reasons; if it was similar, the VR designers might rely on studies which use traditional methods with expectations their findings would also apply to VR, and scientists might use VR methods with the expectation that it will not markedly skew the results. If VR was worse or markedly different somehow, then everyone should be

careful with generalizing across implementations. It has never been our goal to advocate for VR. In our revised introduction we make sure that this is clearer, and include special mention in the last paragraph of the introduction, clarifying the intended purpose of the research.

We also take the reviewers advice and include new discussion of enjoyment and engagement as a possible reason for wanting to favour VR-based methods in research.

At one point the authors pointed out that a virtual task could be used in fMRI research, but one cannot move one's head in the machine, so this does not seem to be a benefit.

>> Immersive VR is being used in a wide variety of research applications, including the neurosciences, which has, as the reviewer notes, significant constraints. Our mention of this research here was only intended to support the idea that despite differences in user interfaces, VR had been found by these researchers to still activate similar cognitive processes to those activated during identical tasks in other modalities.

c. On the flip side of the case, researchers who do not have access to virtual equipment can make the argument that enjoyment of the task (or whatever argument is made for virtual tasks) does not matter – it does not affect performance – and so they can be comfortable using the traditional tasks. This reasoning could be a further benefit for using a virtual task, or it could raise the question of why the current study is important (i.e., why bother?).

>> As described above, it was not our research goal to establish that VR is better. Our contribution provides evidence of similarities and differences across implementations of a category learning experiment, the understanding of which will be useful for both researchers and designers. Our introduction was rewritten to make this more clear, highlighting the implications of either finding

2. The tasks and procedures need more explanation.

a. I did not understand the logistics of what participants did in the tasks. In the 2D tasks I am assuming that they could see all features at a glance, but how was the task presented and how did participants respond? On a desktop computer? Using a joystick? Pencil/paper? Further, what was presented in the 3D condition? The same as in the virtual condition (Figure 4)? How is the virtual controller different than the joystick? If the virtual task used a virtual joystick, it seems that the task itself is identical and only the surrounding environment is different (i.e., looking at the stimuli on a computer in a room vs. being in a room, with the stimuli floating in front of you). Why would this difference have an impact on performance? Again, why bother with a virtual task?

>> We have included additional description that addresses these questions in the Method section for readers less familiar with the category learning paradigm.

b. How many trials were there? In the results section it says that there were 24 trials per bin, implying 240 trials. I am assuming that each category was presented equally, so 60 trials each. Did participants receive 10 (continuous) blocks of trials, each with 4 categories x 6 presentations = 24 trials? How were the trials randomized? This information should be in the methods section.

>> Yes it should; we are sorry for the omission. We have completely revised the Methods section to provide more complete information which answers these questions and others.

c. The dependent measures should be operationalized in more detail. For example, reaction time is measured as the time between the presentation of the cube-to-be-categorized and [what?].

>> We have included additional description of methods, and apologize for not having done a more thorough job in the initial submission.

3. The data analyses need to be more in-depth.
For each measure, there is an initial stage of getting familiar with the task (Bin 1), a learning curve (Bin 2 to asymptotic performance) and an overall difficulty of the task (asymptotic performance, or Bin 10). The learning curve and asymptotic performance should be analyzed separately. This would provide a better comparison of learning differences among the tasks, and whether they are comparable. Any discussion should focus on these more subtle differences among the groups. Here are three examples.

a. Accuracy. Asymptotic performance for all groups appears to be around .90. The VR group appears to reach asymptote at Bin 5, whereas the other groups do not reach asymptote until Bin 10. Surely the learning curve (i.e., slope) differs among the groups? What does this mean? Why would the VR group learn the task more quickly?

>> We did not initially include such analyses because they are not standard for the field, and so any such findings will have no statistically assessed comparison point in existing data. Nevertheless, we have now included additional analyses as requested, which also enlarge the discussion slightly as we consider the implications of these results.

b. Optimization. Asymptotic performance for all groups appears to be around .80. The ET/2D group reaches optimization at Bin 4, whereas the other groups reach it at Bin 6 or 7. What does it mean that the VR and 3D optimize fixations on relevant features at the same rate?

>> Done. We conducted similar block inclusive analyses and found interaction effects for many of the dependent variables, suggesting differing rates of learning and asymptotic performance between groups. These differences are now stated in the Results section. Since the data have been changed (see comment 5 response), the specific differences observed in these comments are no longer applicable.

c. Response Time. The ET/2D group is dramatically faster even in Bin 1. Any further analysis may need to use Bin 1 as a covariate. The ET/2D reaches asymptote at Bin 3 or 4, whereas the other groups reach asymptote at Bin 8 or 9 (if at all). Again, what do the differences and similarities mean in terms of the cognitive processes involved in category learning?

>> As above (3a & b).

4. The number of non-learners is concerning.
I understand that the percentages of non-learners may be similar to previous research, but the number (nearly 50%) is still concerning. What is happening with these participants?

A criterion of 24 consecutive correct trials was set as the inclusion criterion. This means that they had to have reached asymptotic performance to be included. I understand that any model of cognitive processes should include only performance that is accurate. However, even though the non-learners may not have reached asymptotic performance within 240 trials, their learning curves are still informative. A good cognitive model should include when/where there are breakdowns in performance. If their data are too variable, use the median rather than the mean for each bin, where possible. At some point I would like to see the non-learner's data, even if not in this manuscript.

>> Our aim, to assess the link between VR procedures to prior 2D versions necessitated using standard category learning procedures. The category structure on which we based the current work and its associated standard procedures has been known to produce many non-learners, and in choosing this particular procedure, we inherited its associated non-learner rates. Nevertheless, we completely agree with the reviewer's sentiment. We are currently doing experimental work to understand possible reasons these individual differences, but such an evaluation (long past due in our view) goes beyond the present focus on VR, nor is it exclusive to the category structure used here.

To give the reviewer insight into the non-learner data, we include similar plots from the manuscript, except using only non-learners' data below. These plots show the mean and standard error for each bin within the separate groups. As visible in the first figure, the non-learners' accuracy was only moderately better than random chance (25%). The overall mean accuracy for all non-learners was 31.97%. The low accuracy, mostly 0 optimization, low fixation counts, and short feedback durations show patterns of participants who possibly gave up at some point the experiment, or were bored and clicked through trials rapidly in order to finish the experiment faster. The main exception to 0 optimization is the ET condition. The non-learner ET data is rather sporadic due to the equipment failures (as indicated by the large fluctuations in standard error). All bins in which ET participants had perfect or near perfect optimization had an average of two or less fixations, low accuracy (all below 60%, most at 25%), and fast response times, indicating the participants likely looked at one or both of the relevant features due to

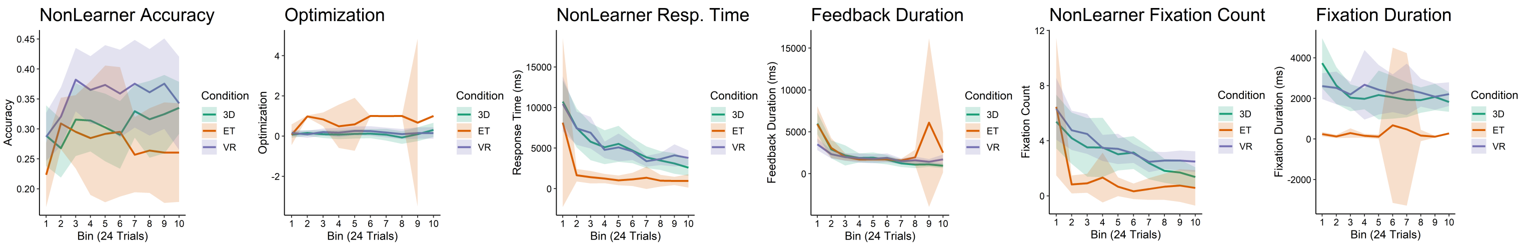

random chance.

5. The inclusion of a 2D/ET group completing a task with different stimuli is concerning. It is unclear whether the differences in performance for the 2D group is because of the task or the stimuli. Either gather more data with the current stimuli or state that there were not enough remaining participants to analyze. Or, you could analyze the data you have. Your data are stable, with each measure in each bin averaged across 24 trials per person. When this is the case, we often only need 8-10 participants per group to find an effect.

>> Following your advice, we have returned to the data collected during our initial data collection period, running our pre-planned analyses on the original data collected, and using the alternative dataset for quality assurance purposes so as to assess the reliability of the data given its smaller sample. Using this data does not change our overall conclusions as there are only minor differences between these two sets of data.

Reviewer #2:
The reviewed paper describes user testing of virtual reality (VR) in the scope of application in learning. VR was compared with 2D and 2D visualization. This topic is undoubtedly current and interesting. I have a few comments on the methodology used, especially its description.

Introduction
- Because I have a background in geospatial sciences, I know of several examples of VR applications in this area, such as teaching geography.
- Some other studies compare desktop VR, and immersive VR created using HMD (e.g., Zhao et al., 2020; ~https://www.tandfonline.com/doi/full/10.1080/13875868.2020.1817925~).

Methods
- None of the participants were excluded due to cybersickness?
>> We have updated our exclusion criteria to specify that some of the non-learners were excluded for dropping out after reporting mild discomfort. 6 participants in the VR condition dropped out for this reason, as did 1 participant in the ET condition as well.

- On the one hand, I understand that equipment failures can occur during the experiment; on the other hand, it seems a bit amateurish.

>> Our recent equipment failures are due to our well-used tools, befitting our extensive experience in eye-tracking and category learning (e.g., Blair, Watson, Walshe & Maj, 2009; Blair, Watson & Meier, 2009; McColeman, Ancell & Blair, 2011; Meir & Blair 2012, Chen, Meier and Blair, 2013; McColeman & Blair; 2013; McColeman et al., 2014; Barnes, Blair, Tupper and Walshe, 2021; Dolguikh, Tracey & Blair, 2021).

- I would recommend describing the symbols that served as stimuli in the experiment. On what basis were they selected? Does their selection have any basis in literature?
>> The symbols are shown in figure 1 of the manuscript. Stimuli in category learning often vary, for example, Shepard, Hovland and Jenkins (1961) use multiple objects; Medin and Schaffer (1978) use shapes of difference colours and numbers; Smith and Minda (1998) use cartoon bugs, and so on. Counterbalancing is important for ensuring minor differences in feature salience do not influence global findings, and the present work takes all the standard precautions.

- Determining the fixations for the 3D and VR conditions seems inappropriate. Even if the user had the cube turned to the given symbol, this does not mean that he did not look in the virtual environment other than this cube wall.

>> The worry is unfounded. In our experiment, fixations in both the VR and 3D condition are determined, in part, by the participants head position (the plane of the main camera is linked to the headset) as well in as rotation of the cube. If participants are looking around at the peripheral environment and not at the cube it will not register as a fixation. The possibility exists, of course, that participants will 'zone out' and fixate but not cognitively process the information (Simons, 1999); however, that possibility exists

equally in all eye-tracking work, and not just in those conditions. Finally, we also note that we have published other work in journal Attention, Perception and Psychophysics that used this terminology (McColeman et al., 2020). Consistency with this published work requires us to retain the current terminology.

- HTC Vive can be combined with an eye-tracking device (HTC Vive Pro Eye headset). Why did not the authors try to take advantage of this opportunity? Eye-tracking in VR is described in detail by, for example, Ugwitz et al. (2022; ~https://doi.org/10.3390/app12031027~).

>> It can. The article you cite (Ugwitz et al., 2022) also describes in detail the limitations of current implementations of eye-tracking in VR. We were cognizant of these limitations when deciding on our equipment needs in 2018, and in the end, chose to use the equipment we had available to us.

- It follows from the above that the term "eye-tracking condition" is not appropriate. I recommend replacing the "2D condition" in all parts of the manuscript (e.g., in the abstract, it is correct).

>> There must be some confusion: the eye tracking condition literally used an eye-tracker - a Tobi x120. We have made significant revisions to the Method section in hopes of reducing the chance of avoiding these kinds of confusions; sorry for the trouble.

- I recommend adding illustrative photography of the participants in all three conditions. For example, it will answer whether they sat or stood during the solved tasks.
>> All participants sat for the experiment per the description in the revised method section. Given that the paper has many figures already, we are reluctant to include more, without additional justification. We do not feel very strongly about this issue however, if the editor is in agreement, we can certainly add those images upon request.

- The terms "VR hand controller" and "standard game controller" are unclear. Better describe what kind of device it is, for example, that it was an HTC Vive controller.
>> Done.

Discussion
- I would discuss the experiment results in the context of information equivalence.
>> "Information equivalence" is not a term used in the category learning literature as far as we are aware, and we are not familiar with its technical use. Given that we do not really know what is intended or how to implement this comment, we await further clarification.
