# Supplementary material for: Comparing virtual reality, desktop-based 3D, and 2D versions of a category learning experiment
Source: PLoS One. 2022 Oct 6;17(10):e0275119. doi: 10.1371/journal.pone.0275119 (PMC9536585; doi:10.1371/journal.pone.0275119)
Supplement: Attachment — Submitted filename: CategoryVR_Aug2022_ResponsestoReviewers.docx [file pone.0275119.s002.docx]

**Category-VR Aug2022 PLoS ONE Revise and Resubmit**

**Responses to Reviewer Comments**

**Reviewer #1:**

In this revision, the authors have been very responsive to the reviewers’ comments. The introduction and methods are much improved. I still have concerns about the presentation of the results though. The main suggestion I have is to reorganize the results to focus on (a) learning performance and (b) information seeking behaviors.

**Response: Agreed. Condensing the results into two primary concepts makes for a much more approachable interpretation of the results.**

In the results section, I think that the overall regression model should be stated first and more concisely, stating that all DVs were analyzed this way.
DV = Bin + Condition + Bin* Condition + Bin-squared + Bin-squared*Condition

**Response: Done.**

Separate the six DVs in terms of those that are measures of learning performance and those that are measures of the information seeking behaviors. Presumably, accuracy and response times are learning-related, and fixation count, fixation duration, attentional optimization, and feedback	duration are related to information seeking.

**Response: Sub-headers have been implemented across the paper to better distinguish variables relating to learning outcomes and text relating to information access behaviors.**

A main effect of Bin (or Bin-squared) is expected with all DVs, indicating that performance improves with practice. The main question is whether there is a main effect of condition, and, more importantly, whether condition interacts with the linear and/or quadratic effects of Bin. So, the results should focus on these interactions.

**Response: Done.**

Organizing the information in terms of learning performance and information-seeking behaviors should be reflected in the introduction, methods, and discussion, as well as in the results section. Be sure to use consistent terms throughout the paper for “information-seeking behaviors” – sometimes they are referred to as information access, information sampling strategies, information sampling, or attentional optimization.

**Response: We have settled on “information access behaviors” as the preferred term and have made additional effort to distinguish this from our measure of attentional optimization.**

Other suggestions follow:

a. Include a photo or drawing of the controllers in the three conditions.

**Response: We have added photos of the controllers used in each condition: Fig 3**

b. What are the outcome measures used in the Hamilton et al. meta-analysis? (line 152)

**Response: We have made the quantitative outcome measures reported in this meta analysis more explicit.**

c. The comparison group is missing in several places (e.g., measurable improvements over what?) (lines 153, 165, 178, 186, 226, 237, 243)

**Response: Thank you for pointing this out. We have updated these lines to be clearer.**

d. Add the DVs to Conditions in the Methods section (line 290); for example, “Conditions and Outcome Variables” but save how they were calculated for later in the methods section, in a section called “Calculation of Dependent Measures” (or something like that). “Detecting Fixations” would become part of this new section.

**Response: Done.**

e. The paragraph describing a trial (lines 342-353) is still confusing. It should be in the form: A trial began with_____, then the stimulus appeared, and the participant manipulated the object (VR and 3D conditions) or simply looked at the stimulus (2D condition). When they decided which category the stimulus belonged to, they _____. After responding, they received feedback until they were ready for the next trial, at which time they pressed ____. This produced a arrow with the words “next trial.” When the participant pressed ____, the next trial began.

**Response: We have restructured our procedures description to be more concise using aspects of the template provided by the reviewer here.**

**Reviewer #2:**

Most of my suggestions have been responded to in the manuscript and commented on in the cover letter. The structure of the paper has been improved, and the description of the methodology has been improved. I do not understand why the authors insist on naming the eye-tracking ("ET") condition. J still believe that this tested variant should be marked as "2D".

**Response: We have come (albeit slowly!) to agree with you and the other reviewer regarding the naming of this variable and have changed the name in the manuscript to 2D.**

**Finally, in your response to our initial and most recent revisions, Reviewer #2 indicated that our data is inaccessible online. However, our data availability statement clearly indicates where you can access our publicly released data in .csv format. This link works for us. If you are having any issues accessing this data, please let us know and we can troubleshoot appropriately.**
